# Genome-resolved metagenomic analysis of Great Amazon Reef System sponge-associated Latescibacterota bacteria and their potential contributions to the host sponge and reef

**DOI:** 10.3389/frmbi.2023.1206961

**Published:** 2023-08-17

**Authors:** Rafael S. Oliveira, Otávio H. B. Pinto, Betania F. Quirino, Mayanne A. M. de Freitas, Fabiano Lopes Thompson, Cristiane Thompson, Ricardo H. Kruger

**Affiliations:** ^1^Laboratory of Enzymology, Department of Cell Biology, Institute of Biological Sciences, University of Brasília, Brasília, Brazil; ^2^Genetics and Biotechnology Laboratory, Embrapa-Agroenergy, Brasília, Brazil; ^3^Genomic Sciences and Biotechnology Graduate Program, Catholic University of Brasília, Brasília, Brazil; ^4^Microbial Biology Graduate Program, University of Brasília, Brasília, Brazil; ^5^Department of Genetics, Institute of Biology, Federal University of Rio de Janeiro, Rio de Janeiro, Brazil

**Keywords:** great amazon reef system (GARS), host-associated, metagenome-assembled genomes (MAGs), sponge microbiome, turbid reef

## Abstract

The Great Amazon Reef System (GARS) is an extensive biogenic reef influenced by a plume layer of sediments. This creates an extreme environment where light is reduced, thus affecting physicochemical properties as well as living organisms such as sponges and their microbiomes. The sponge’s microbiome has numerous ecological roles, like participation in biogeochemical cycles and host nutrition, helping the sponge thrive and contributing to the ecosystem. Also, sponges and sponge-associated microorganisms are rich sources of bioactive compounds, and their products are applied in different areas, including textile, pharmaceutical, and food industries. In this context, metagenome-assembled genomes (MAG), obtained from GARS sponges microbiota, were analyzed to predict their ecological function and were prospected for biotechnological features. Thus, in this work, tissues of GARS sponges were collected, their metagenomes were sequenced and assembled, and 1,054 MAGs were recovered. Ten of those MAGs were selected based on their taxonomic classification in the candidate phylum Latescibacterota and this group’s abundance in GARS sponges. The workflow consisted of MAG’s quality definition, taxonomic classification, metabolic reconstruction, and search for bioactive compounds. Metabolic reconstruction from medium to high-quality MAGs revealed genes related to degradation and synthesis pathways, indicating functions that may be performed by GARS sponge-associated Latescibacterota. Heterotrophy, a recurring attribute in Latescibacterota that might be crucial for GARS sponge holobiont nutrition, was verified by the presence of genes related to respiration and fermentation. Also, the analyzed bacteria may contribute to the host’s survival in multiple ways, including host protection via defense systems; aid in nutrient consumption by breaking complex substrates and producing essential nutrients like vitamins and certain amino acids; and detoxification of mercury, arsenic, ammonia, and hydrogen sulfide. Additionally, genes linked to persistent organic pollutant degradation, including glyphosate, and biogeochemical cycles reactions, such as ammonification, sulfate reduction, thiosulfate disproportionation, phosphorus remineralization, and complex organic matter degradation, were identified, suggesting the participation of these Latescibacterota in bioremediation and nutrient cycling. Finally, the investigated MAGs contain genes for numerous bioactive compounds, including industrial enzymes, secondary metabolites, and biologically active peptides, which may have biotechnological value.

## 1 Introduction

For many years, elevated concentrations of suspended sediments and low light levels were considered threats to marine invertebrates’ growth. These conditions might affect sponges by reducing their growth or disturbing their physiology, morphology, and microbial community, resulting in increased bleaching and mortality (Pineda et al., 2017; Zweifler et al., 2021). However, there is currently enough evidence to support that marine invertebrate communities can live in natural turbid reefs and benefit from these circumstances by becoming more resilient to stress and bleaching. To thrive in an environment with high sediment load, turbid sponge communities use strategies to rapidly adapt to these conditions, such as reducing channel openings, raising mucus production and photoefficiency, or using heterotrophy to compensate for reduced photosynthesis (Pineda et al., 2017; Burt et al., 2020; Zweifler et al., 2021). An example of a turbid reef is the Great Amazon Reef System (GARS).

The GARS is an extensive biogenic reef located near the Amazon River mouth, between the Brazil-Guyana border and the Brazilian state of Maranhão (latitude: 5° N to 1° S; longitude: 44° W to 51° W), with an estimated area of 9,500 km^2^ and a depth of 220 m (Moura et al., 2016; Francini-Filho et al., 2018). The GARS is composed of multiple habitats that shelter a diversity of species, including algae, sponges, corals, crustaceans, and fish (Moura et al., 2016). The region is greatly influenced by the Amazon River, which discharges up to 300 × 10^3^ m^3^/s of water rich in suspended matter. The massive amount of water and suspended sediments discharged alters physicochemical properties of the place, including pH, salinity, dissolved nutrients, and lighting (Mahiques et al., 2019). Additionally, this discharge generates a plume containing dissolved and particulate nutrients, sediments, and microbes. The GARS plume attenuates light, changes nitrogen, and organic matter availability, and may provide nutrients for local sponges (Moura et al., 2016; Francini-Filho et al., 2018; Menezes et al., 2022).

Sponges are sessile aquatic filter feeder invertebrates that can be considered a holobiont formed by the host sponge and its associated organisms (Hentschel et al., 2012; Pita et al., 2018). These associated organisms are found inside or outside cells, usually in the mesohyl, where a diverse microbial community lives (Taylor et al., 2007; Bibi et al., 2017). This microbiome, which may correspond to 40% of the sponge’s total volume, includes viruses, bacteria, archaea, and unicellular eukaryotes (Webster and Taylor, 2012). Sponge microbial communities are highly diverse and comprise numerous species of bacteria, including cultivated bacteria, uncultivated bacteria, and members of candidate phyla (KIran et al., 2018). Interactions between microorganisms and the host sponge can be either beneficial or detrimental to the participants. Microorganisms can help the sponge acquire nutrients and protect it against predators and pathogens. On the other hand, the symbionts get a supply of nutrients, obtained during water filtration, as well as shelter from predators and light (Taylor et al., 2007; Selvin et al., 2010). Conversely, microorganisms can harm the sponge by directly acting as a parasite or pathogen, causing damage to its skeletal structure, or indirectly via biofilm formation that promotes surface encrustation, resulting in channel blockage and displacement. On the other side of the interaction, sponges produce inhibitory and selective substances that shape their microbiome and might consume symbionts for nutrition (Taylor et al., 2007; Thacker and Freeman, 2012). Furthermore, sponge-associated microbes are involved in several ecological processes. They can participate in biogeochemical cycles, including carbon, nitrogen, sulfur, and phosphorus, and impact ecological relations, affecting predation and competitiveness (KIran et al., 2018; Pita et al., 2018).

Sponges and sponge-associated microorganisms are rich sources of biologically active compounds. They produce various classes of secondary metabolites such as peptides, polyketides, terpenes, alkaloids, pigments, sterols, and fatty acids, which exhibit relevant properties like antimicrobial, antiparasitic, antitumor, anticancer, anti-inflammatory, antioxidant, and cytotoxic (Bramhachari et al., 2016; Bibi et al., 2017; Brinkmann et al., 2017). The sponge holobiont is also a valuable source of hydrolytic enzymes that transform organic matter into nutrients. Many of these biocatalysts, including amylase, protease, cellulase, lipase, and esterase, are applied in the textile, pharmaceutical, and food industries. Sponge-derived enzymes occasionally show unique characteristics like thermal, acid, alkaline, and salinity tolerance (Santos-Gandelman et al., 2014). The sponge holobiont has immense biotechnological potential, but due to difficulties in large-scale production, few sponge-derived compounds have progressed to clinical stage or have been approved for use (Brinkmann et al., 2017). Culture-independent methods, such as metagenomics and metatranscriptomics, can be applied to access the biotechnological potential of hard-to-isolate microorganisms and obtain genes to efficiently produce a desired molecule (Pallela and Kim, 2011). Another application of sponge symbionts concerns bioremediation, which involves the conversion of toxic substances, such as oils and heavy metals, into less damaging forms by microorganisms (Abatenh et al., 2017). The sponge’s microbiome can also indicate the presence of contaminants and be used as a pollution bioindicator via changes in community composition or the detection of resistance genes (Selvin et al., 2010; Santos-Gandelman et al., 2014).

The candidate phylum Latescibacterota is a group of uncultured bacteria composed of heterotrophic microorganisms. This candidate phylum has been identified in the microbial communities of waters and animals, including marine invertebrates (Vavourakis et al., 2018; Engelberts et al., 2020). In this work, 10 Latescibacterota metagenome-assembled genomes, obtained from bacteria associated with GARS sponges, were investigated to predict their ecological role and potential contributions to the host and the reef, helping to explain how these sponges thrive in a light-reduced habitat. Additionally, knowing that the GARS is a unique location with peculiar characteristics and sponge microbiomes are rich sources of bioactive compounds, biotechnological features were searched for in the MAGs.

## 2 Materials and methods

### 2.1 Sample collection and DNA extraction

Sponge samples were collected near the mouth of the Amazon River (latitude: 1.299817; longitude: -46.778867) on September 27, 2014. Samples from sponges of the *Agelas* and *Geodia* genera were collected, including the species *Agelas dispar*, *Agelas clathrodes*, *Agelas clathrodes*, *Geodia* sp., *Geodia* cf. *corticostylifera*, and *Geodia neptuni*. The main criterion for sponge selection was the availability of at least three individuals per genus. After being sampled on the RV, the sponges were immediately stored in liquid nitrogen. Each sponge’s tissue was dried and dissected with a scalpel to remove associated macroscopic organisms. Approximately 1 g of sponge tissue was frozen in liquid nitrogen and subsequently pulverized. DNA from a single individual of a selected sponge species was extracted and purified with 4 M guanidine hydrochloride, 50 mM Tris-HCl (pH 8.0), 0.05 M EDTA, 0.5% sodium N-lauroylsarcosine, and 1% β-mercaptoethanol. This was followed by phenol/chloroform extraction, isopropanol precipitation, and resuspension in 50 µL of ultrapure water (Trindade-Silva et al., 2012).

### 2.2 Metagenome sequencing and assembly

A DNA library was constructed for each sample with Illumina® TruSeq Nano DNA Preparation Kit. Sequencing was performed on the Novaseq system (Illumina®), generating 150 bp paired-end reads at 20 GB sequence coverage (**Supplementary Data Set S1**). Sickle v1.33 (https://github.com/najoshi/sickle) trimmed the reads, while BBtools v35 (https://sourceforge.net/projects/bbmap/) identified and removed phiX sequences and Illumina adapters. A total of six metagenomes were generated, one for each sponge species. The metagenomes were assembled with metaSPAdes v3.13.0 (Nurk et al., 2017), removing scaffolds shorter than 1 kb. All programs were used with default settings.

### 2.3 MAGs recovery and selection

Metagenome-assembled genomes (MAGs) were recovered using MaxBin2 v2.2.14 with 40 markers for bacteria and archaea, and 107 markers for bacteria only (Wu et al., 2016). DASTool v1.1.2 aggregated the MAGs (Sieber et al., 2018). uBin v0.9.14 (Bornemann et al., 2023) selected MAGs based on GC content, coverage, and taxonomy. Dereplication was executed with dRep v3.2.2 (Olm et al., 2017) with a threshold of 95%. Default parameters were used in all of these programs. A total of 1,054 MAGs were recovered from the sponges’ microbiome. To exclude low-quality MAGs, coverage and contamination were calculated, respectively, by CoverM v0.6.1 (https://github.com/wwood/CoverM) and CheckM v1.0.13 (Parks et al., 2015). Genomes with <70% completeness or >10% contamination were discarded.

The remaining 205 MAGs underwent additional analysis. These MAGs were annotated with Prokka v1.14.5 (Seemann, 2014) and taxonomically assigned using the SILVA database v138 of 16S rRNA genes (Quast et al., 2013). MAGs with inconclusive 16S rRNA classification results were classified using phylogenetic marker genes trees. Based on these preliminary classifications, 10 MAGs were selected for further investigation as they were all assigned to the same under-described group, Latescibacterota. This phylum was selected because it is abundantly found in GARS sponges with low microbial abundance (Menezes et al., 2022), and its members may represent up to 2% of the GARS sponges’ metagenomes (Pinto et al., 2022) (**Supplementary Data Set S2**). Moreover, Latescibacterota members cannot be cultivated yet; therefore, one method of learning more about this candidate is through analysis of their genomes.

### 2.4 Genomes description

The quality of selected genomes was more accurately assessed based on criteria established by Bowers et al. (2017), including completeness, contamination, presence of 5S, 16S, and 23S rRNA genes, and the quantity of tRNA. CheckM, CoverM, Prokka, and GenomeQC web server (Manchanda et al., 2020) were used to obtain these genomic metrics. Additional assembly statistics that help define MAG quality, such as the number of scaffolds, assembly size, N50, L50, NG50, LG50, and GC content, were acquired with GenomeQC.

Furthermore, eukaryotic-like proteins (ELP), which contain domains important for symbiosis, were searched using Pannzer2 and CD-Search web servers in default settings. Pannzer2 uses the Uniprot database, and these proteins were manually identified using the description of annotated genes (Törönen and Holm, 2022). CD-Search utilizes a specific conserved domain database (CDD v3.20), and ELPs were manually curated according to the domain name (Marchler-Bauer and Bryant, 2004).

### 2.5 Taxonomic assignment and genome comparison

GTDB-Tk (Chaumeil et al., 2020; version 2.3.0) assigned the selected MAGs to a taxonomic group using GTDB (Parks et al., 2020; version 214). A codon tree was generated using resources present in PATRIC v3.6.12 (Davis et al., 2020). The “Similar Genome Finder” function found genomes similar to the MAGs, while the “Phylogenetic Tree” function identified homologous groups of single-copy genes, aligned those sequences, and concatenated the alignments. The tree was constructed using maximum likelihood estimation with RAxML (Stamatakis, 2014). FigTree v1.4.4 (https://github.com/rambaut/figtree) was used to visualize the tree, making aesthetic changes for improved visualization. The codon tree was composed of Latescibacterota members, members from a similar phylum (Gemmatimonadetes), and an outgroup of Firmicutes that was used to root the tree.

Additionally, average nucleotide index (ANI) and digital DNA-DNA hybridization (dDDH) were used to compare the 10 MAGs and determine their relatedness. While ANI was calculated on JspeciesWS v.3.9.1 (Richter et al., 2016), dDDH was calculated on GGDC v2.1 (Meier-Kolthoff et al., 2013). Default settings were applied to both web servers.

### 2.6 Metabolic reconstruction

Metabolic reconstruction was performed by verifying the existence of genes involved in reactions of specific metabolic pathways in KEGG v97.0 (Kanehisa et al., 2017) and Metacyc v26.0 (Caspi et al., 2020) databases. BlastKOALA v2.2 (Kanehisa et al., 2016) and KEGG Mapper v5.0 (Kanehisa and Sato, 2020) were used to reannotate genomes and access KEGG data. The software Pathway Tools v23.0 was used to reconstruct pathways present in Metacyc (Karp et al., 2021). Graphics and tables were generated with LibreOffice Calc and LibreOffice Writer, while the R package “pheatmap” was utilized to create heat maps.

### 2.7 Biotechnological prospection

Genes that encode industrial enzymes were searched for in annotated genomes. The enzymes were selected based on previously reported applications in scientific papers, using the BRENDA database (Jeske et al., 2019) to aid in the selection (**Supplementary Data Set S3**). AntiSMASH v6.0 (Blin et al., 2021) was used to identify clusters of genes related to the production of secondary metabolites. DbCAN2 web server (Zhang et al., 2018) searched for carbohydrate-active enzymes (CAZyme) present in CAZy database v08062022 (Drula et al., 2022) and dbCAN HMMdb v11 using three tools: Hmmer, Diamond, and eCAMI. Only the genes identified by these three methods were considered valid.

Additionally, biologically active peptides (BAP) were searched for. Peptide sequences were extracted from annotated genomes using FaBox tools v1.61 (Villesen, 2007). Due to software limitations, only sequences with <100 amino acid residues were retrieved. PeptideRanker calculated the chance that a BAP would be present in a protein sequence. Sequences with a probability >0.5 were selected for functional prediction. AntiCP 2.0 (Agrawal et al., 2021), Antifp server (Agrawal et al., 2018), antiAngioPred server (Ramaprasad et al., 2015), and Toxinpred server (Gupta et al., 2013) predicted, respectively, anticancer, antifungal, anti-inflammatory, and toxin activities. Antimicrobial activity was also predicted using antiBP2 server (Lata et al., 2010) and CAMPr3 (Waghu et al., 2016). A predicted function was considered valid when the calculated probability value was >0.5. All programs were run with default settings.

## 3 Results

### 3.1 Genomes description

According to the criteria defined by Bowers et al. (2017), a high-quality genome draft must have 5S, 16S, and 23S rRNA genes, at least 18 tRNA genes, completeness >90%, and contamination <5%. Medium-quality drafts only need completeness ≥50% and contamination <10%. Finally, low-quality drafts have completeness <50% and contamination >10%. Therefore, only three of the selected metagenome-assembled genomes (MAGs) are considered high-quality genome drafts (**Supplementary Data Set S4**). The others are medium-quality genomes due to contamination above 5%, completeness less than 90%, or the absence of certain rRNA genes. Additional metrics used to define MAG quality were also obtained and are described in **Supplementary Data Set S4**.

**Figure 1:**
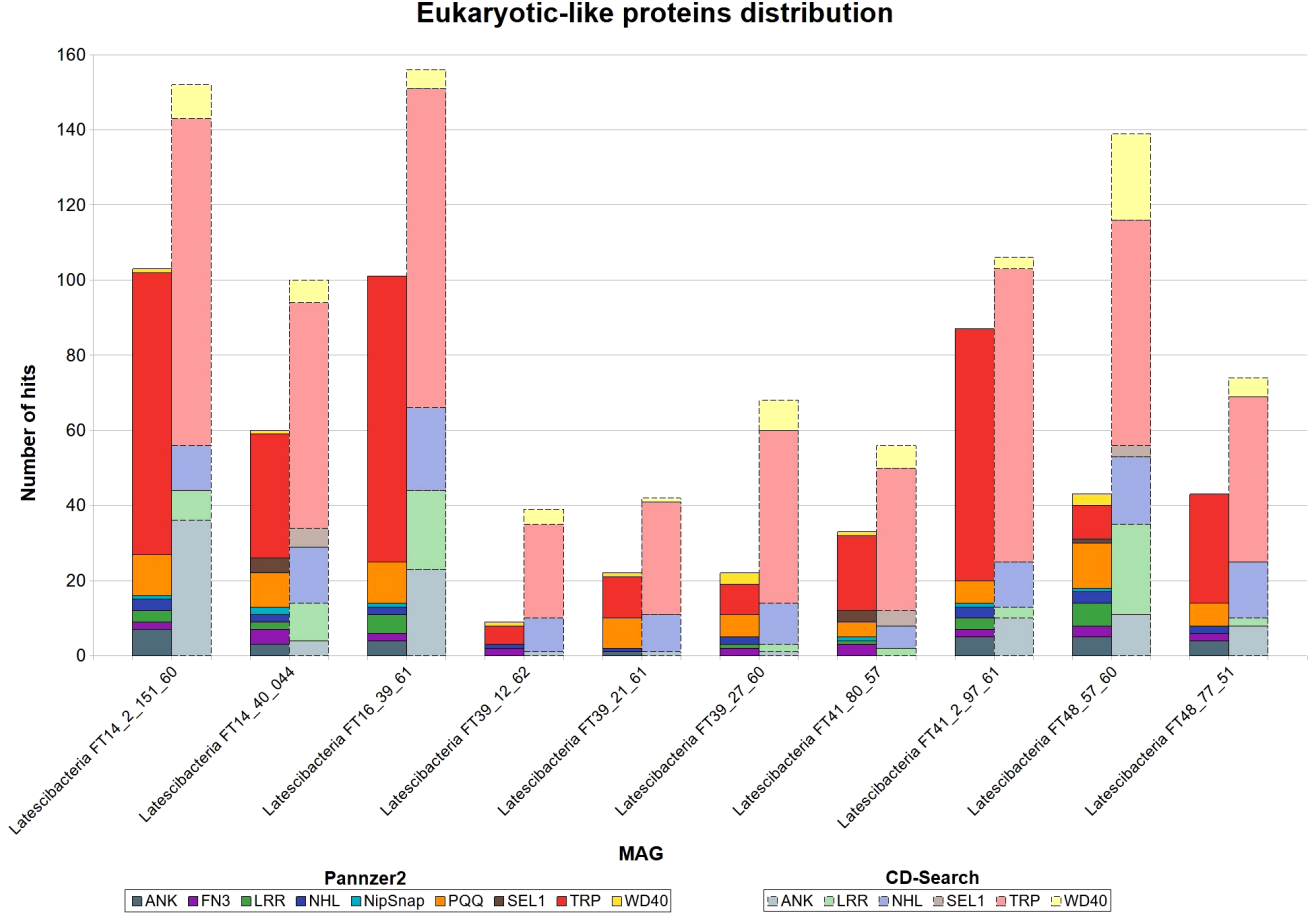
Eukaryotic-like proteins distribution in 10 GARS sponge-associated Latstescibacterota MAGs. Results of eukaryotic-like proteins (ELP) identification in each GARS sponge-associated Latescibacterota MAGs, using the programs CD-Search and Pannzer2. Straight bars depict Pannzer2 results while dashed bars depict CD-Search results. ANK, Ankyrin repeat; FN3, Fibronectin type-III repeat; LRR, Leucine-rich repeat; NHL, NCL-1, HT2A and Lin-41 repeat; NipSnap, NipSnap family; PQQ, Pyrrolo-quinoline quinone; SEL1, Sel1-like repeat; TRP, Tetratricopeptide repeat; WD40, WD40 repeat.

Both Pannzer2 and CD-Search detected several copies of eukaryotic-like proteins (ELPs) genes in all MAGs (**Figure 1**). The following domains were found in CD-Search: ankyrin repeat (ANK); leucine-rich repeat (LRR); NCL-1, HT2A, and Lin-41 repeat (NHL); SEL1-like repeat (SEL1); tetratricopeptide repeat (TPR); and WD40 repeat (WD40). Pannzer2 identified the same domains found by CD-Search, with the addition of fibronectin type III domain (FN3), pyrroloquinoline quinone repeat (PQQ), and NipSnap family. For both programs, the tetratricopeptide repeat (TRP) was the predominant ELP.

### 3.2 Taxonomic classification

**Figure 2:**
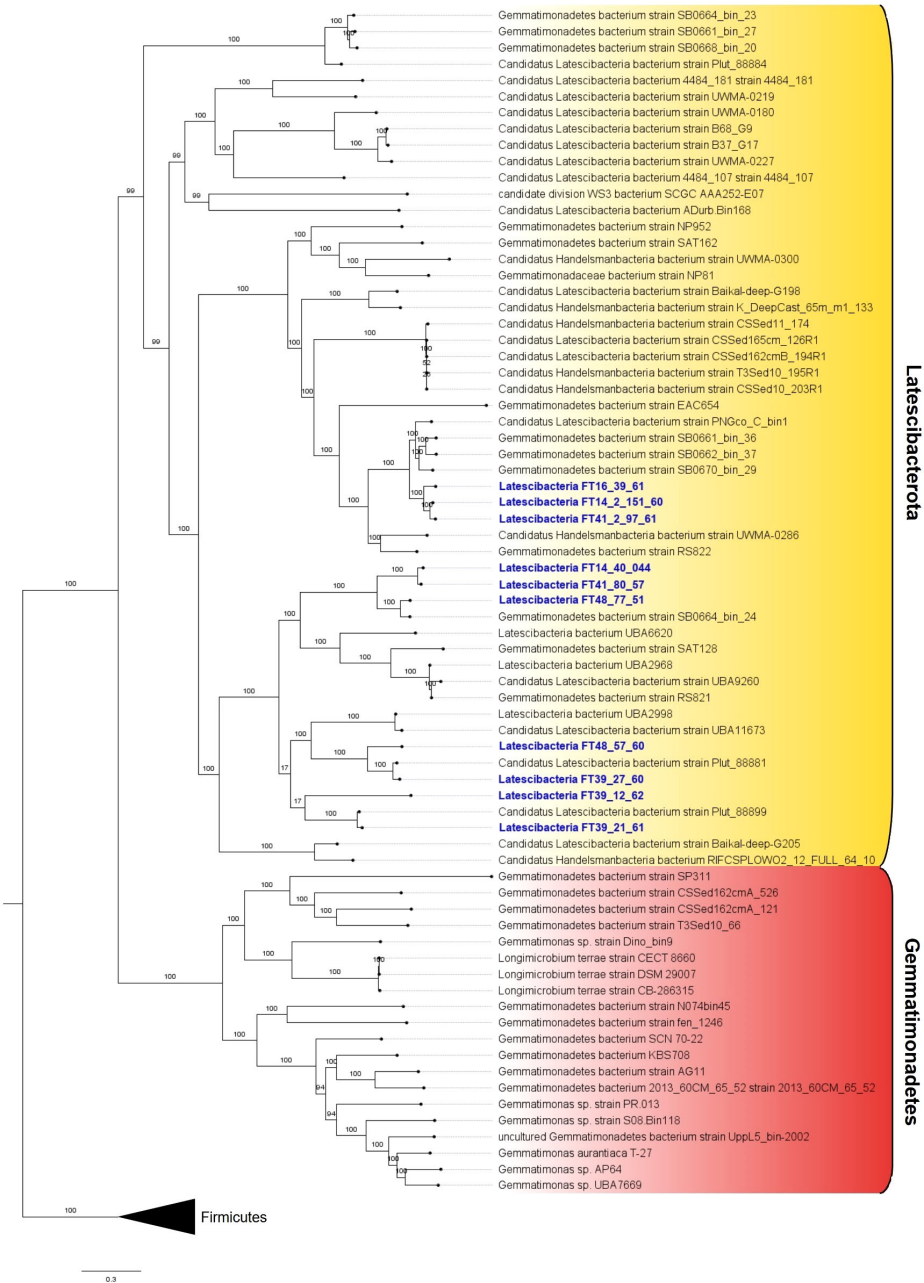
Phylogenetic tree with 10 GARS sponge-associated Latstescibacterota MAGs. Codon tree showing the 10 analyzed GARS sponge-associated MAGs (in blue) in relation to Latescibacterota candidate phylum members (in yellow background) and the similar phylum Gemmatimonadetes (in red background). The tree was rooted in the outgroup composed of Firmicutes members.

All 10 MAGs showing inconclusive taxonomic affiliation based on 16S rRNA genes were assigned to the candidate phylum Latescibacterota by GTDB-Tk (**Supplementary Data Set S4**). The codon tree shows the relation between the selected MAGs, other individuals from the candidate phylum Latescibacterota, and members from the close-related phylum Gemmatimonades (**Figure 2**).

In addition, ANI and dDDH were used to assess how similar the genomes were to one another. ANI values between 95% and 96% or dDDH values ≥70% indicate that the pair of analyzed genomes probably belong to the same species (Chun et al., 2018). Thus, the pair of MAGs Latescibacteria FT14_2_151_60/Latescibacteria FT41_2_97_61 (ANI >96%; dDDH =73.6%) and Latescibacteria FT41_80_57/Latescibacteria FT14_40_044 (ANI >95%; dDDH =72.3%) are likely from the same species (**Table 1**).

**Table 1:** Nucleotide-level genomic similarity comparison between candidate phylum Latescibacterota MAGs by Average nucleotide index (ANI) and digital DNA-DNA hybridization (dDDH) .

		Latescibacteria FT14_2_151_60	Latescibacteria FT14_40_044	Latescibacteria FT16_39_61	Latescibacteria FT39_12_62	Latescibacteria FT39_21_61	Latescibacteria FT39_27_60	Latescibacteria FT41_80_57	Latescibacteria FT41_2_97_61	Latescibacteria FT48_57_60	Latescibacteria FT48_77_51
ANI	Latescibacteria FT14_2_151_60	*	67.4%	90.71%	64.72%	65.32%	64.84%	66.73%	**96.01%**	66.13%	66.32%
	Latescibacteria FT14_40_044	67.56%	*	68.23%	65.69%	67.62%	66.33%	**95.62%**	66.99%	66.83%	77.32%
	Latescibacteria FT16_39_61	90.84%	68.04%	*	64.71%	65.41%	64.95%	67.03%	90.97%	66.08%	66.64%
	Latescibacteria FT39_12_62	64.89%	65.54%	64.71%	*	69.65%	67.64%	65.84%	64.83%	67.71%	64.8%
	Latescibacteria FT39_21_61	65.47%	67.43%	65.7%	69.66%	*	70.04%	67.38%	65.58%	70.48%	66.98%
	Latescibacteria FT39_27_60	66.34%	66.25%	65.4%	67.53%	70.12%	*	66.39%	65.37%	81.03%	66.1%
	Latescibacteria FT41_80_57	66.47%	**96.44%**	66.95%	65.78%	67.43%	66.57%	*	66.12%	66.76%	77.88%
	Latescibacteria FT41_2_97_61	**96.89%**	67.12%	91.69%	64.7%	65.46%	64.88%	66.23%	*	65.99%	66.15%
	Latescibacteria FT48_57_60	65.86%	66.75%	65.94%	67.57%	70.36%	80.58%	66.64%	65.79%	*	66.65%
	Latescibacteria FT48_77_51	66.07%	77.85%	66.51%	65.23%	67.31%	65.94%	77.74%	66.01%	66.63%	*
dDDH	Latescibacteria FT14_2_151_60	*	25.5%	43.2%	21.7%	23.2%	16.9%	24.6%	**73.6%**	22.7%	21%
	Latescibacteria FT14_40_044	25.5%	*	24.8%	17.2%	21.6%	15.3%	**72.3%**	27.9%	18.2%	22.2%
	Latescibacteria FT16_39_61	43.2%	24.8%	*	22.4%	24.8%	21.4%	28%	45.7%	19.2%	24.7%
	Latescibacteria FT39_12_62	21.7%	17.2%	22.4%	*	18.9%	16.6%	28.9%	29.9%	18.1%	23.7%
	Latescibacteria FT39_21_61	23.2%	21.6%	24.8%	18.9%	*	19.2%	19.4%	22.4%	18.4%	28.3%
	Latescibacteria FT39_27_60	16.9%	15.3%	21.4%	16.6%	19.2%	*	15.7%	15.7%	24.8%	16.4%
	Latescibacteria FT41_80_57	24.6%	**72.3%**	28%	28.9%	19.4%	15.7%	*	23.6%	15.4%	22.2%
	Latescibacteria FT41_2_97_61	**73.6%**	27.9%	45.7%	29.9%	22.4%	15.7%	23.6%	*	19.7%	27.9%
	Latescibacteria FT48_57_60	22.7%	18.2%	19.2%	18.1%	18.4%	24.8%	15.4%	19.7%	*	21.9%
	Latescibacteria FT48_77_51	21%	22.2%	24.7%	23.7%	28.3%	16.4%	22.2%	27.9%	21.9%	*

Values above the cutoff (≥95% for ANI; ≥70% for dDDH) are highlighted in black background.

*, 100% match.

### 3.3 Metabolic reconstruction

**Figure 3:**
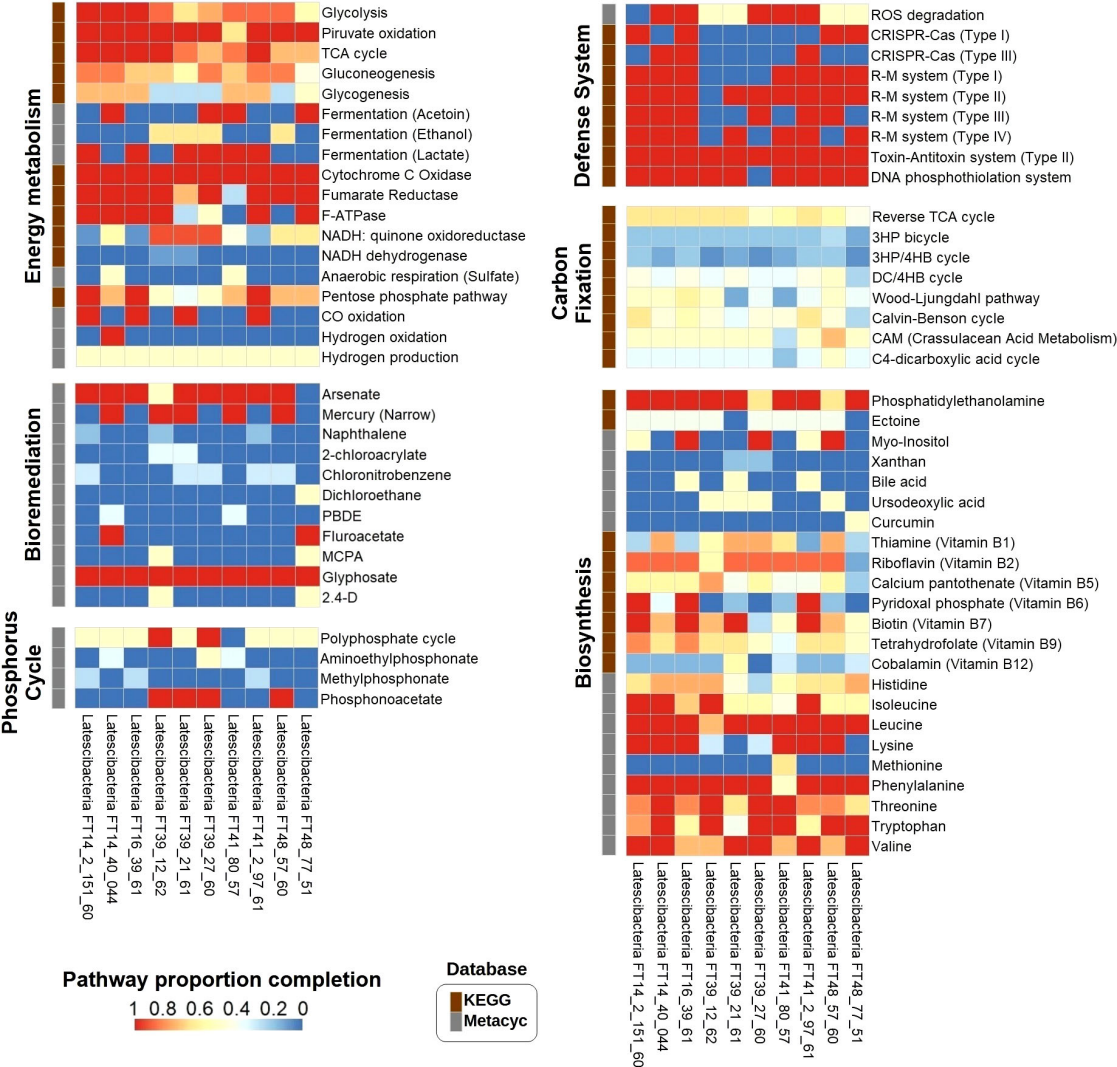
Proportion completion of pathways in 10 GARS sponge-associated Latstescibacterota MAGs. Heat map showing the proportion completion of different pathways in each GARS sponge-associated Latstescibacterota MAG based on the presence of specific genes involved in a certain pathway. The described pathways are related to energy metabolism, carbon fixation, bioremediation, biosynthesis, phosphorus cycle, and defense mechanisms. Information about the pathways were obtained from KEGG (brown squares) or Metacyc (grey squares) databases. 3HP, 3-hydroxypropionate; 4HB, 4-hydroxybutyrate; DC, Dicarboxylate; PBDE, Polybrominated diphenyl ether; MCPA, 2-methyl-4-chlorophenoxyaceticacid; 2,4-D, 2,4-dichlorophenoxyacetic acid.

**Supplementary Data Set S5** contains a list of all the genes and potentially expressed proteins that were identified during metabolic reconstruction, including transporters and enzymes. All Latescibacterota MAGs contained complete heterotrophic aerobic processes like the tricarboxylic acid (TCA) cycle, the electron transport chain (complexes I–V), and ATP production (**Figure 3**). As for anaerobic processes, a gene related to the dissimilatory sulfate reduction to hydrogen sulfide (*sat*) was present in seven MAGs, whereas genes related to dissimilatory reduction of nitrate to ammonium (*narI* and *nirD*) were present in six MAGs. Genes for pyruvate fermentation to acetoin (*alsS* and *budC*), ethanol (*adh*), or lactate (*ldh*) were found in all MAGs. Glycolysis and the pentose phosphate pathway were among the complete catabolic processes observed in three MAGs. Moreover, all of the analyzed Latescibacterota have the gene that encodes the enzyme carbon monoxide dehydrogenase, linked to energy generation from carbon monoxide. Furthermore, the gene for hydrogenase, an enzyme used to generate energy from hydrogen, was found in the Latescibacteria FT14_40_044 MAG. Finally, genes for carbon fixation pathway enzymes were found in all MAGs, though all those pathways are incomplete and genes for key enzymes are missing (**Figure 3**).

In addition, metabolic reconstruction results indicate that GARS sponge-associated Latescibacterota may degrade a variety of organic and inorganic molecules, including carbohydrates, lipids, amino acids, and other sulfur, phosphorous, and nitrogen compounds (**Supplementary Data Set S5**). The findings also suggest that they can synthesize important molecules such as carbohydrates, lipids, phospholipids, essential amino acids, vitamins, and secondary metabolites (**Figure 3**). Complete pathways for the production of phosphatidylethanolamine, myo-inositol, vitamins B6 and B7, and the essential amino acids isoleucine, leucine, lysine, phenylalanine, threonine, thryptophan, and valine were identified.

**Figure 4:**
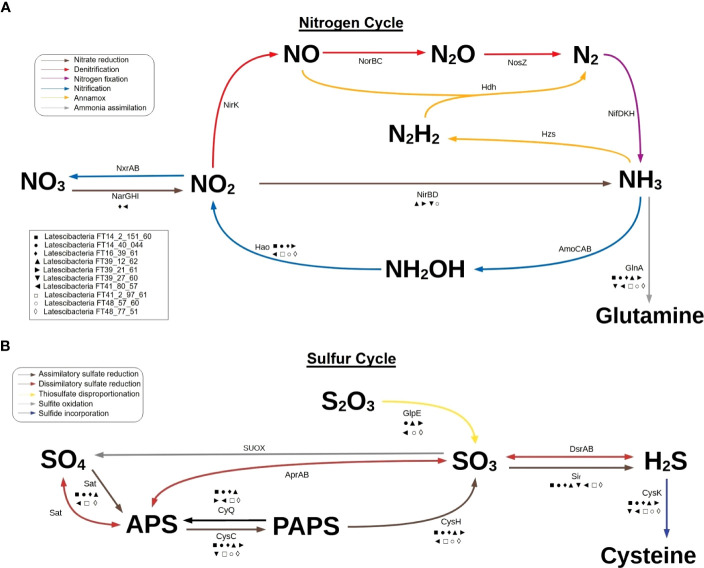
Participation of 10 GARS sponge-associated Latescibacterota MAGs in nitrogen cycle and sulfur cycle. Predicted involvement of GARS sponge-associated bacteria from the candidate phylum Latescibacterota in biogeochemical cycles based on the presence of specific genes in MAGs. **(A)** Representation of the nitrogen cycle highlighting the possible participation of GARS Latescibacterota in nitrate reduction, nitrification, and ammonia assimilation. **(B)** Representation of the sulfur cycle, emphasizing the possible participation of GARS Latescibacterota in assimilatory sulfate reduction, dissimilatory sulfate reduction, thiosulfate disproportionation, and sulfide incorporation. Each of the 10 MAGs analyzed is represented by a symbol, as shown in the box. The presence of a MAG symbol below an arrow indicates that the gene involved in the reaction is present in the MAG.

Furthermore, the MAGs contain genes for enzymes involved in specific steps of the nitrogen, sulfur, and phosphorus cycles. Regarding the nitrogen cycle, genes for hydroxylamine oxidation to nitrite (*hao*), nitrate reduction to nitrite (*narI*), and conversion of nitrite to ammonia (*nirD*) were detected (**Figure 4A**). These genes indicate participation in nitrification, interconversion of nitrate and nitrite, and ammonification. Two genes required for the incorporation of ammonia into amino acids during glutamine synthesis (*glnA* and *gltB*) were observed as well. Concerning the sulfur cycle, the results suggest participation in assimilatory sulfate reduction (*sat*, *cysC*, *cysH*, and *sir*), dissimilatory sulfate reduction (*sat*), thiosulfate disproportionation (*glpE*), and incorporation of sulfide into cysteine (*cysK*) (**Figure 4B**). As for the phosphorus cycle, metabolic reconstruction indicates a contribution to phosphorus remineralization due to the presence of genes related to organic phosphorus compound degradation, such as aminoethyl phosphonate (*phnW* and *phnY*), methyl phosphonate (*phnPP*), and phosphonoacetate (*phnA*). Additionally, all genes for the polyphosphate cycle (*ppx*, *ppk*, and *ppk2*), a pathway involved in phosphorus storage, were verified in two MAGs (**Figure 3**).

Moreover, MAGs analysis shows that GARS sponge-associated Latescibacterota might be involved in heavy metals metabolism (**Figure 3**). All genes for arsenic compound bioremediation (*arsC* and *gap*) and narrow mercury resistance (*merA* and *merP)* were detected in eight and five MAGs, respectively. These microorganisms might also participate in the degradation of harmful organic compounds such as naphthalene, 4-chloronitrobenzene, 1,2-dichloroethane, fluoroacetate, and polybrominated diphenyl ether (PBDE), as verified respectively by the identification of genes *nagAB*, *cnbZ*, *dhmA*, *dehH2*, and *bphA*. Additionally, all MAGs contain the gene for glycine oxidase (*thiO*), which acts in the bioremediation of the pesticide glyphosate, whereas two MAGs have the gene for 2,4-dichlorophenol 6-monooxygenase (*tdfA*), which is involved in the degradation of the herbicides 2,4-dichlorophenoxyacetic acid (2,4-D) and 2-methyl-4-chlorophenoxyacetic acid (MCPA).

Importantly, the following prokaryotic defense mechanisms were observed in the GARS sponge-associated Latescibacterota MAGs: clustered regularly interspaced short palindromic repeats (CRISPR) and CRISPR-associated protein (Cas) system (types I and III); restriction-modification (R-M) system (types I, II, III, and IV); toxin-antitoxin system (type II); and DNA phosphothiolation system (**Figure 3**). Five MAGs also contain the catalase (katG) and superoxide dismutase (sodA) genes, which encode enzymes that degrade reactive oxygen species (ROS).

Lastly, genes that encode enzymes related to the production of antibiotics were present in all Latescibacterota sponge-associated MAGs (**Supplementary Data Set S5**). However, all pathways were incomplete. The enzymes found were associated with the production of aurachin (*auaCH*), anthracycline (*dauE*), bacilisin (*bacCG*), beta-lactams (*axeA*, *acyII*, *cefD*, *G7AC*, *PENDE*), fosfomycin (*fom1*), kanamycin (*kanJ*), lividomycin (*livQ*), macrolide (*pikAII*), novobiocin (*novN*), pentalenolactone (*ptlD*), prodigiosin (*pigC*), pyocyanin (*phzF*), pyrrolnitrin (*prnD*), tetracycline (*oxyT*), and chlortetracycline (*ctcP*). Antibiotic resistance genes were also found in the 10 MAGs, indicating resistance to multiple drugs, including aminoglycosides, macrolides, vancomycin, penicillin, and triclosan (**Supplementary Data Set S5**).

### 3.4 Biotechnological prospection

The GARS sponge-associated Latescibacterota MAGs contain genes that encode industrial enzymes, including lipase, phospholipase, esterase, protease, aminopeptidase, amidase, dioxygenase, monooxygenase, dehalogenase, nitrile hydratase, alginate-lyase, alcohol dehydrogenase, and glycerol dehydrogenase (**Table 2**).

**Table 2:** Enzyme-coding genes with biotechnological potencial identified in candidate phylum Latescibacterota MAGs from GARS sponges’ microbiome.

Enzyme	EC Number	Latescibacteria FT14_2_151_60	Latescibacteria FT14_40_044	Latescibacteria FT16_39_61	Latescibacteria FT39_12_62	Latescibacteria FT39_21_61	Latescibacteria FT39_27_60	Latescibacteria FT41_80_57	Latescibacteria FT41_2_97_61	Latescibacteria FT48_57_60	Latescibacteria FT48_77_51
Alcohol dehydrogenase	EC1.1.1.2										
Glycerol dehydrogenase	EC1.1.1.6										
Catalase	EC1.11.1.6										
Hydrogenase	EC1.12.-.-										
Dioxygenase	EC1.13.12.-										
Monooxygenase	EC1.14.13.-										
Esterase	EC3.1.1.-										
Lipase	EC3.1.1.3										
Phospholipase	EC3.1.1.32										
Alkaline phosphatase	EC3.1.3.1										
Epoxide hydrolase	EC3.3.2.9										
Protease	EC3.4.-.-										
Glutaminase	EC3.5.1.2										
Amidase	EC3.5.1.4										
Dehalogenase	EC3.8.1.-										
Nitrile hydratase	EC4.2.1.84										
Alginate lyase	EC4.2.2.-										

Green squares indicate the presence of a gene that encodes a certain enzyme in a MAG. Red squares indicate gene**’**s absence.

**Figure 5:**
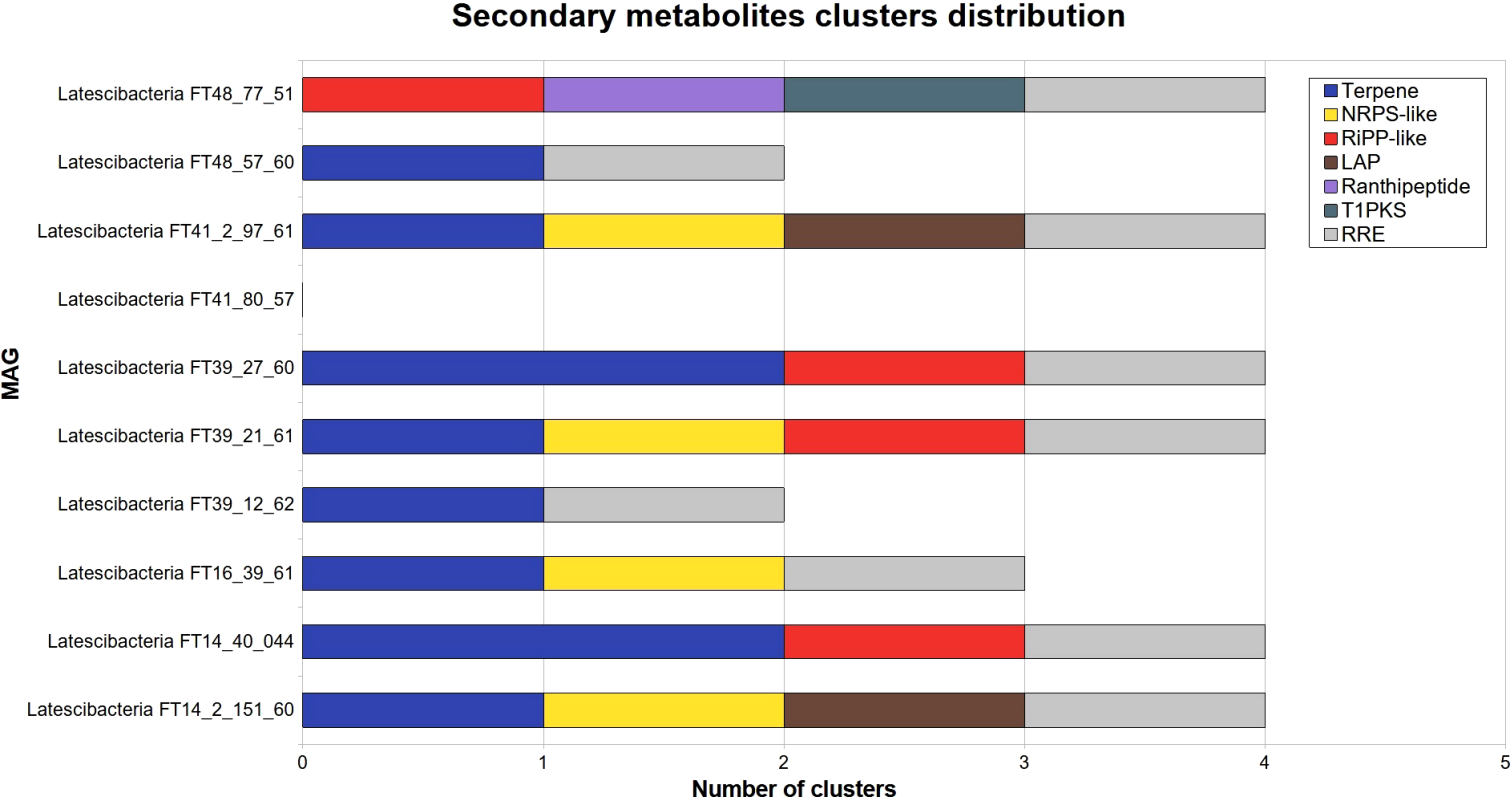
Secondary metabolites clusters distribution in 10 GARS sponge-associated Latescibacterota MAGs. Identification of clusters of genes related to secondary metabolites production in GARS sponge-associated Latescibacterota MAGs, according to AntiSMASH. These clusters were absent in Latescibacteria FT41_80_57. The following types of secondary metabolites clusters were identified: Non-ribosomal peptide synthetase-like fragment (NRPS-like); other unspecified ribosomally synthesized and post-translationally modified peptides (RiPP); linear azol(in)e-containing peptides (LAP); type I polyketide synthase(T1PKS); and RiPP recognition element containing cluster (RRE).

Gene clusters involved in secondary metabolites production were identified by AntiSMASH in all GARS sponge-associated Latescibacterota MAGs, with the MAG Latescibacteria FT41_80_57 as an exception (**Figure 5**). The clusters are linked to the production of terpene, non-ribosomal peptide synthase-like fragment (NRPS-like), linear azole(in)e-containing peptide (LAP), polyketide synthase type I (T1PKS), ranthipeptide, cluster containing RiPP recognition element (RRE), and other unspecified ribosomally synthesized and post-translationally modified peptide product (RiPP-like).

**Figure 6:**
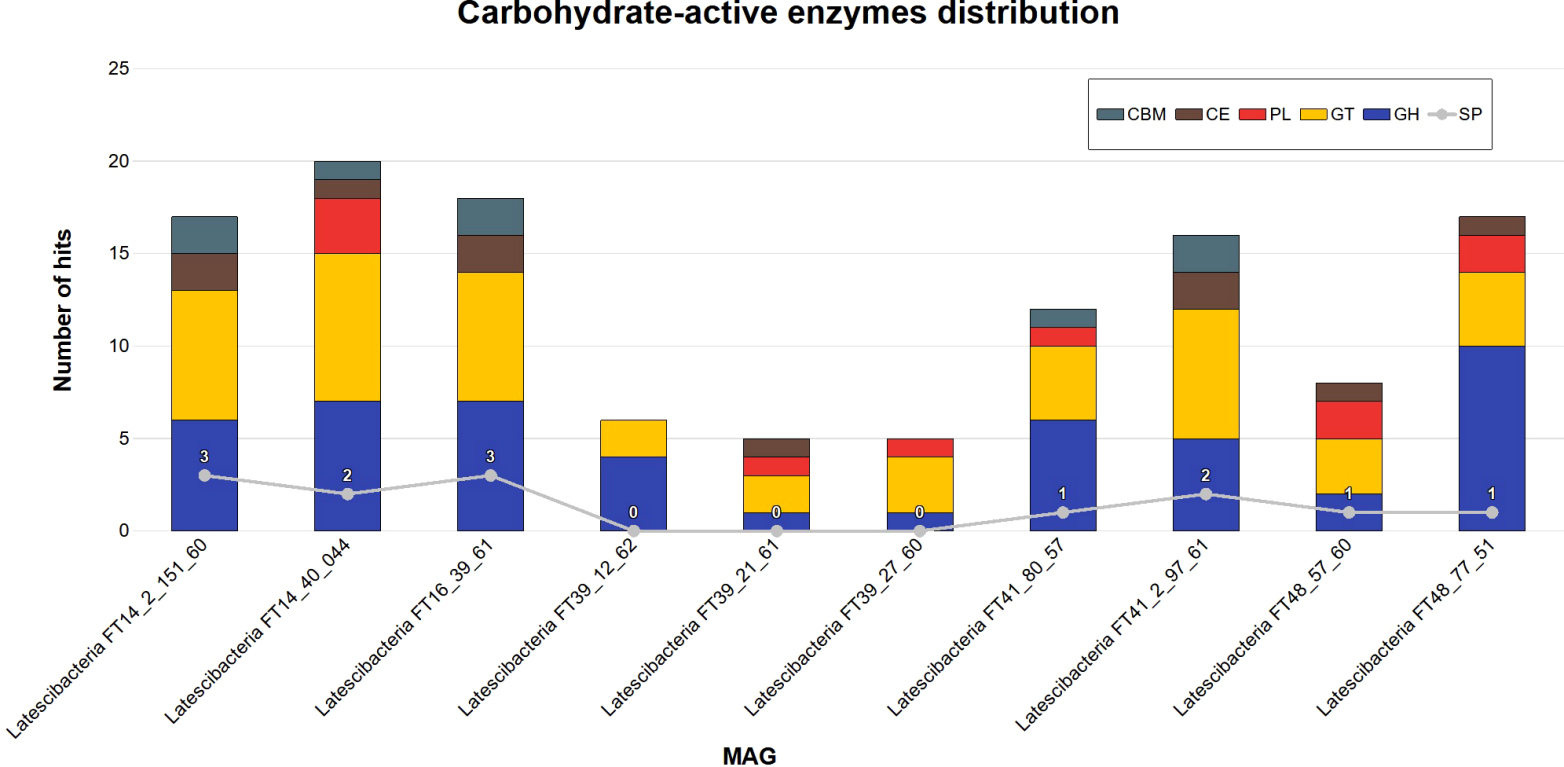
Carbohydrate-active enzymes distribution in 10 GARS sponge-associated Latescibacterota MAGs. Five distinct classes of carbohydrate-active enzymes (CAZyme) were identified in GARS sponge-associated Latescibacterota MAGs by DbCAN2: Carbohydrate-binding modules (CBM); Carbohydrate esterases (CE); Polysaccharide lyases (PL); GlycosylTransferases (GT); Glycoside hydrolase (GH). The auxiliary activities class was absent. 13 sequences also contained a signal peptide (SP).

A total of 124 genes for CAZymes were annotated by DbCAN2 in the 10 MAGs (**Figure 6**). The following modules were identified: glycoside hydrolase (GH); glycosyltransferase (GT); polysaccharide lyase (PL); carbohydrate esterase (CE); and carbohydrate-binding modules (CBM). The auxiliary activity module (AA) was absent. It is worth noting that 13 sequences also have a signal peptide.

**Figure 7:**
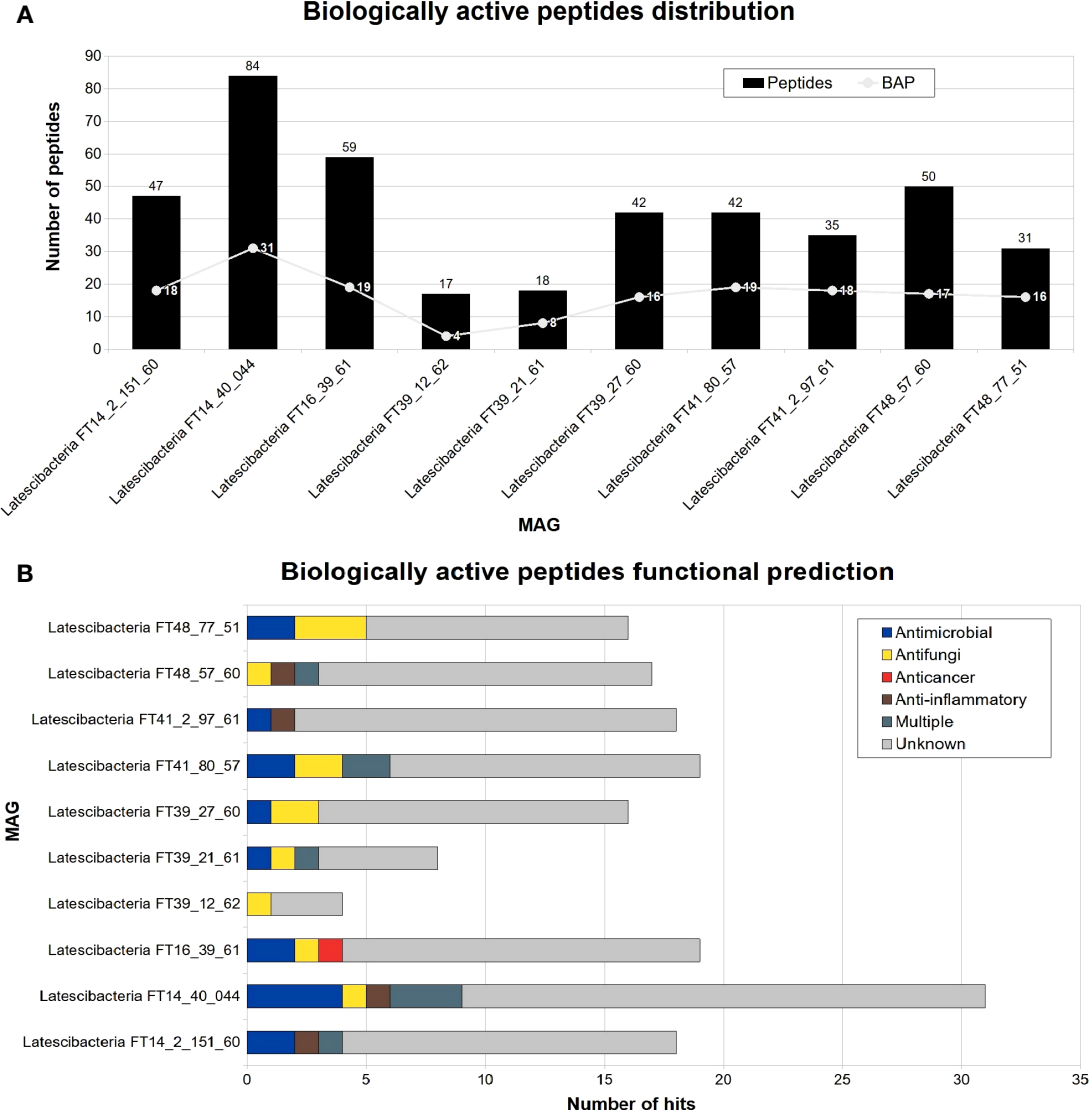
Peptides distribution and functional prediction in 10 GARS sponge-associated Latescibacterota MAGs. Number of biologically active peptides (BAP) predicted in each GARS sponge-associated Latescibacterota MAGs. **(A)** Total number of peptides (i.e., amino acid sequences with less than 100 residues) and number of peptides with a predicted biological function. **(B)** Function of predicted biologically active peptides.

A total of 425 peptides were identified in all sponge-associated Latescibacterota MAGs. Of those peptides, 166 were categorized as biologically active peptides (BAP) by PeptideRanker (**Figure 7A**). In 126 of those BAPs, there was no biological function predicted. Nevertheless, antimicrobial, antifungal, anti-inflammatory, and anticancer activities were predicted in 40 BAPs (**Figure 7B**). For eight peptides, more than one type of activity was predicted. In contrast, Toxinpred did not detect toxin activity in any peptide.

## 4 Discussion

### 4.1 Contributions to the sponge and symbiosis

GARS MAGs provide valuable insights into the functional potential of uncultured Latescibacterota associated with the sponge holobiont. These MAGs encode enzymes that degrade complex substrates such as proteases and lipases, releasing nutrients for easier absorption by the sponge (Selvin et al., 2010; Pita et al., 2018). These bacteria also possess transporters that may transfer nutrients and other compounds between the host and symbionts (Fan et al., 2012; Menezes et al., 2022). Additionally, they can synthesize myo-inositol, which serves as an osmolyte or part of cell structures, and phosphatidylethanolamine, which is a structural component of sponges and bacterial membranes (Murzyn et al., 2005; Roberts, 2006; Genin et al., 2008). Moreover, these microorganisms could supply the host’s demand for essential nutrients like vitamins and some amino acids (Pita et al., 2018; Robbins et al., 2021).

Another role potentially performed by the GARS sponge-associated Latescibacterota is host defense. Their MAGs contain genes involved in the production of antibiotics, biologically active peptides, and other secondary metabolites with antibiotic activity, which can prevent pathogen colonization (Selvin et al., 2010). Secondary metabolites may also contribute to chemical defense, predator evasion, and physiological improvement, influencing the sponge’s susceptibility to predation (Pita et al., 2018). Additionally, the 10 MAGs have genes related to stress proteins, defensive enzymes like phospholipases, and defense systems such as CRISPR-Cas, R-M system, toxin-antitoxin system, and DNA phosphotiolation. These genes, which are generally enriched in sponge symbionts, may protect the sponge against harmful substances and foreign organisms (Selvin et al., 2010; Horn et al., 2016). Moreover, five MAGs contain genes for antioxidant enzymes, which may improve the host’s defense against reactive oxygen species by increasing antioxidant activity and neutralizing capability (Regoli et al., 2000).

Furthermore, GARS sponge-associated Latescibacterota could aid in the elimination or mitigation of toxic substances. They may oxidize and assimilate ammonia, one of the main wastes generated by sponges, preventing its accumulation during moments of low water pumping and the consequential death of host tissues (Webster and Taylor, 2012; Pita et al., 2018). These bacteria may also regulate the concentration of hydrogen sulfide, which can be toxic in aquatic environments, by incorporating sulfide into cysteine, similar to the process observed in plants (Bagarinao, 1992; Birke et al., 2012). In addition, GARS sponge-associated Latescibacterota potentially convert mercury and arsenic into less toxic forms, safeguarding the host from their harmful effects. This protection could be crucial as heavy metal exposure impacts the sponge’s microbial community, physiology, and overall health (Selvin et al., 2010; Webster and Taylor, 2012).

To establish a symbiotic relationship, sponges can recognize and differentiate symbionts from consumable microbes through immune signaling (Thomas et al., 2010). ELPs, which are usually enriched in sponges and their symbionts, may play a role in this recognition as they are linked to phagocytosis evasion (Pita et al., 2018). Different ELPs were identified in the GARS sponge-associated Latescibacterota, consistent with findings in other sponge-associated microorganisms (Reynolds and Thomas, 2016; Díez-Vives et al., 2017; Frank, 2019).

### 4.2 Contributions to the reef

Besides aiding their host, GARS sponge-associated Latescibacterota can contribute to the reef by participating in nitrogen, sulfur, and phosphorus cycles. Concerning the nitrogen cycle, genes related to the interconversion of nitrate and nitrite, ammonification, nitrification, and incorporation of ammonia into amino acids were identified. Nitrifying bacteria and ammonia-oxidizing bacteria are frequently found in sponges from different environments (Pajares and Ramos, 2019). About the sulfur cycle, the analyzed Latescibacterota might be involved in assimilatory sulfate reduction, dissimilatory sulfate reduction, and thiosulfate disproportionation. However, this last reaction may serve a different purpose as it is catalyzed by rhodanese, an enzyme used to convert hydrogen cyanide into less toxic forms (Cipollone et al., 2007). Nonetheless, these Latescibacterota are likely sulfate-reducing bacteria, a type of microorganism regularly found in association with sponges (Jensen et al., 2017; Lavy et al., 2018). Regarding the phosphorus cycle, the described Latescibacterota may participate in phosphorus storage through its sequestration and deposit in polyphosphate granules, which are produced during the polyphosphate cycle. This process affects phosphorus availability as well as marine microbial metabolism and diversity (Dyhrman et al., 2007; Zhang et al., 2015; Pita et al., 2018). There is also possible involvement in remineralization via phosphonate degradation, a pathway commonly found in invertebrates’ microbiomes (Villarreal-Chiu et al., 2012; Podell et al., 2020).

The investigated microorganisms might also contribute to bioremediation. GARS sponge-associated Latescibacterota MAGs contain genes related to the degradation of pollutants such as naphthalene, 4-chloronitrobenzene, 1,2-dichloroethane, organohalides, fluoroacetate, PBDE, glyphosate, 2,4-D, and MCPA. However, none of these contaminants directly impact sponges. Conversely, PBDE is produced by a few species of sponges (Agarwal et al., 2017). Nonetheless, some of these pollutants, including naphthalene, glyphosate, and certain types of PBDE, are known persistent organic pollutants in the ocean, disturbing other aquatic organisms (Ipen, 2018; Vagi et al., 2021). Moreover, all MAGs contain genes for bioremediation enzymes like oxygenases, nitrile hydratase, and dehalogenase, which can break, transform, or remove toxic molecules such as aromatics, nitriles, and organohalogens (Karigar and Rao, 2011; Cheng et al., 2020; Oyewusi et al., 2020).

Furthermore, metabolic reconstruction of GARS sponge-associated Latescibacterota revealed an energy metabolism typical of heterotrophic bacteria, with coexisting aerobic and anaerobic processes. Heterotrophy is a feature often found in bacteria associated with sponges, typically through aerobiosis. However, facultative anaerobes and sulfate-reducing bacteria can be found in sponges’ microbiomes, possibly due to pumping fluctuations that create moments of anaerobiosis (Osinga et al., 2001; Taylor et al., 2007). In the GARS, heterotrophic processes may be important for sponges, particularly the ones with low microbial abundance, as they can obtain a carbon supply from organic particles in the Amazon River plume (Calegario et al., 2021; Menezes et al., 2022). Carbon fixation is another process occurring in the GARS turbid areas, albeit at a minor level (Pinto et al., 2022). These pathways are frequently seen in sponge symbionts and the candidate phylum Latescibacterota (Feng and Li, 2019; Tran et al., 2021), but were absent or incomplete in the 10 MAGs. Carbon monoxide dehydrogenase, an enzyme frequently present in litohererotrophics, was detected in all MAGs, which may allow the use of carbon monoxide as a carbon source or for energy conservation (Robb and Techtmann, 2018; Burgsdorf et al., 2022).

### 4.3 MAG’s quality and limitations

It is worth mentioning that the MAGs’ quality varied from medium to high. The assembly quality is essential for annotation and may affect the quantity and quality of identified genes (Florea et al., 2011), which could explain the predominance of incomplete pathways in the studied MAGs. Another hypothesis that could explain this incompleteness of metabolic pathways is the presence of alternative genes encoding unknown enzymes that may be involved in the missing reactions (Lannes et al., 2019). In some pathways, cooperation with other organisms in the community may be necessary to complete metabolic processes. Through the metabolic division of labor, different populations execute complementary reactions of a particular pathway, as seen in nitrification (Embree et al., 2015; Tsoi et al., 2018). Microorganisms can also interact with the host through cosynthesis or cometabolism. In sponges, integration between symbiont and host genes may be required to complete a pathway, or the product of one organism’s reactions may act as an input in the other’s pathway (Selvin et al., 2010). It is important to highlight that inferences were made using DNA sequences and did not include information about gene expression. Having a specific gene in a MAG does not guarantee that a particular process is being executed. Instead, it suggests potential for its execution.

### 4.4 Biotechnological prospection

In addition to their ecological role, GARS sponge-associated Latescibacterota may serve as a significant source of natural products as they potentially produce relevant metabolites. For example, these bacteria may produce myo-inositol, which is used in the food, pharmaceutical, and cosmetic industries as a beverage additive as well as to produce uronic acid and other inositol derivatives (You et al., 2020). Moreover, nine MAGs contain clusters of genes involved in the generation of secondary metabolites, including terpenes, ranthipeptides, LAPs, T1PKS, and NRPS-like proteins, as well as clusters with RRE, a conserved domain that interacts with peptides and is used to identify novel compounds (Burkhart et al., 2015). Sponge symbionts are a known source of NRPS and PKS (Pimentel-Elardo et al., 2012; Trindade-Silva et al., 2013). Except for ranthipeptides, whose function remains unknown, these metabolites may have exploitable activities. For instance, terpenes are employed as a repellent, pigment, flavoring agent, and medical component for infections and cardiovascular diseases (Schempp et al., 2018; Cox-Georgian et al., 2019).

Additionally, genes for commercial enzymes that are applied in many areas were identified in GARS sponge-associated Latescibacterota MAGs. For instance, proteases are used in the food industry to process meat, milk, and beverages, as well as in the production of pharmaceuticals, agrochemicals, and textiles (Adrio and Demain, 2014; Raveendran et al., 2018). Esterases, phospholipases, and aminopeptidases are also used in the food industry to enhance food flavor and fragrance (Raveendran et al., 2018; Nandan and Nampoothiri, 2020). Moreover, five classes of CAZymes, enzymes that act on the synthesis, breakdown, or modification of complex carbohydrates and other glycoconjugates, were found in the 10 MAGs (Cantarel et al., 2009). CAZymes from marine organisms are used in several areas, including bioenergy, food industry, and chemical industry, and may possess unique properties (Oliveira et al., 2020; Contesini et al., 2021).

Furthermore, GARS sponge-associated Latescibacterota showed great potential for generating bioactive peptides, containing possible peptides with antimicrobial, antifungal, anticancer, and anti-inflammatory properties predicted. BAPs are a group of peptides with less than 50 amino acid residues that exhibit a biological function (Macedo et al., 2021; Akbarian et al., 2022). These peptides also have significant pharmaceutical and nutraceutical potential, with commercialized peptide products derived from sponges such as the antibacterial polydiscamide A and the anticancer phakellistatin (Brinkmann et al., 2017; Macedo et al., 2021).

In conclusion, the 10 sponge-associated Latescibacterota may play a significant role in the sponge’s success in the GARS. These symbionts may participate in the host’s nutrition and protection against pathogens, heavy metals, and other harmful chemicals. Also, the presence of genes related to biogeochemical cycles and bioremediation indicates that these Latescibacterota contribute to nutrient flux in the reef and mitigation of damage from detrimental compounds. Additionally, the predominance of heterotrophic processes is in agreement with a recent hypothesis that GARS organisms might feed from sediments and other particles present in the Amazon River discharge (Menezes et al., 2022). However, it is essential to acquire transcriptional data to confirm the expression of found genes and endorse the execution of discussed functions. Finally, GARS sponge-associated Latescibacterota have great biotechnological value. Their genomes contain genes for industrial enzymes, secondary metabolites, and biologically active compounds that could be explored in future works.

## Data availability statement

The datasets presented in this study can be found in online repositories. The names of the repository/repositories and accession number(s) can be found below: https://www.ncbi.nlm.nih.gov/genbank/, The sequence datasets generated for this study were deposited at NCBI under Bio-Project number PRJNA795684.

## Ethics statement

Ethical review and approval was not required for the study on animals in accordance with the local legislation and institutional requirements.

## Author contributions

RO, OP, and RK designed the study. Data analyses were performed by OP and RO. Data collection was performed by FT. The manuscript was drafted by RO and revised by all authors. BQ, MF, FT and CT critically reviewed and substantially edited the manuscript. All authors contributed to the article and approved the submitted version.
